# Supplementary material for: Lethal Arrhythmogenic Role of Left Ventricular Myocardial Interstitial Fibrosis in Apolipoprotein E/Low-Density Lipoprotein Receptor Double-Knockout Mice with Metabolic Dysfunction-Associated Steatohepatitis
Source: Int J Mol Sci. 2024 Dec 27;26(1):144. doi: 10.3390/ijms26010144 (PMC11720108; doi:10.3390/ijms26010144)
Supplement: Supplementary file 1 [file ijms-26-00144-s001.zip › ijms-3271718-supplementary.pdf]

Table S1. Breeding results data bank

ID	Group	BW (g)	SCD or AD		Ethanol		LV (g)	liver (g)
			total consumption (g)	daily consumption (g/week/Kg BW)	total consumption (g)	weekly consumption (g/week/Kg BW)		
1	AL+SCD, Et ( )	Died during rearing						
2	AL+SCD, Et ( )	30	459.5	1078.7			0.112	1.14
3	AL+SCD, Et ( )	27	491.5	1115.1			0.101	0.883
4	AL+SCD, Et ( )	32	488	1019.2			0.083	1.157
5	AL+SCD, Et ( )	32	431	977.2			0.103	1.156
6	AL+SCD, Et ( )	27.5	426.5	1072.4			0.098	1.13
7	AL+SCD, Et ( )	30	479.5	1130.5			0.113	1.26
8	AL+SCD, Et ( )	28	422.5	956.2			0.1	1.093
9	AL+SCD, Et ( )	Died just before the lethal arrhythmia-evoked test						
10	AL+AD, Et ( )	50	464	707.7			0.153	2.57
11	AL+AD, Et ( )	49	504	831.6			0.192	2.555
12	AL+AD, Et ( )	41	405	719.6			0.135	2.103
13	AL+AD, Et ( )	45	444	763.7			0.136	2.4
14	AL+AD, Et ( )	Died during rearing						
15	AL+AD, Et ( )	45.5	405.5	646.8			0.131	2.092
16	AL+AD, Et ( )	41.5	420.5	1192.8			0.168	1.76
17	AL+AD, Et ( )	41	421.5	676.2			0.126	1.94
18	AL+AD, Et ( )	26	577.5	1239.0			0.118	1.31
19	AL+AD, Et (+)	31.5	364.5	783.3	70.3	149.1	0.187	1.784
20	AL+AD, Et (+)	42	356.5	649.6	69.3	119.0	0.167	2.439
21	AL+AD, Et (+)	35	354.5	748.3	64.1	126.7	0.2	*
22	AL+AD, Et (+)	Died the day before the end of the study						
23	AL+AD, Et (+)	42.5	376	614.6	78.0	123.2	0.142	*
24	AL+AD, Et (+)	43	378.5	639.8	72.2	121.8	0.166	2.75
25	AL+AD, Et (+)	35.5	352	559.4	55.4	85.9	0.17	3.89
26	AL+AD, Et (+)	41	395	654.0	53.8	87.9	0.158	1.98
27	WT+SCD, Et ( )	32.5	460.5	1001.7			0.112	0.112
28	WT+SCD, Et ( )	33	483.5	1038.8			0.114	0.114
29	WT+SCD, Et ( )	32	497.5	1100.4			0.114	0.114
30	WT+SCD, Et ( )	32.5	463.5	1034.6			0.114	0.114
31	WT+SCD, Et ( )	34	466.8	1009.4			0.118	0.118
32	WT+SCD, Et ( )	32.5	448	991.2			0.102	0.102
33	WT+SCD, Et ( )	31.5	412	984.9			0.112	0.112
34	WT+SCD, Et ( )	32	416	957.6			0.113	0.113

*: The mice died during the lethal arrhythmia-evoked test, resulting in the inability to collect liver samples from these individuals.

Table S2. Blood ethanol concentration results data bank

ID	Group	total ethanol consumption (g)	weekly consumption (g/week/Kg BW)	Blood ethanol concentration (mg/mL)	blood storage location
19	AL+AD, Et (+)	70.3	149.10	Blood collection failure	
20	AL+AD, Et (+)	69.3	119.00	0.18	box1
21	AL+AD, Et (+)	64.1	126.70	Died during ARS.	
22	AL+AD, Et (+)	Died the day before the end of the study			
23	AL+AD, Et (+)	78	123.20	Died during ARS.	
24	AL+AD, Et (+)	72.2	121.80	0	box1
25	AL+AD, Et (+)	55.4	85.89	0	box1
26	AL+AD, Et (+)	53.8	87.85	0.12	box1

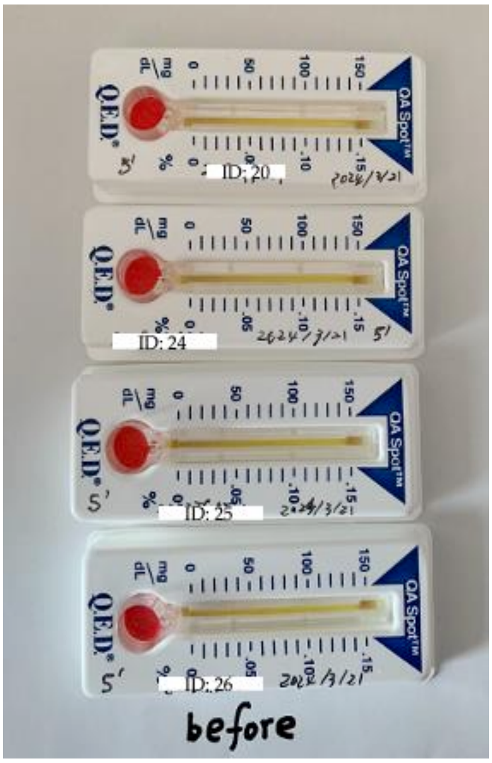

Figure S1 Immediately before adding blood sample.

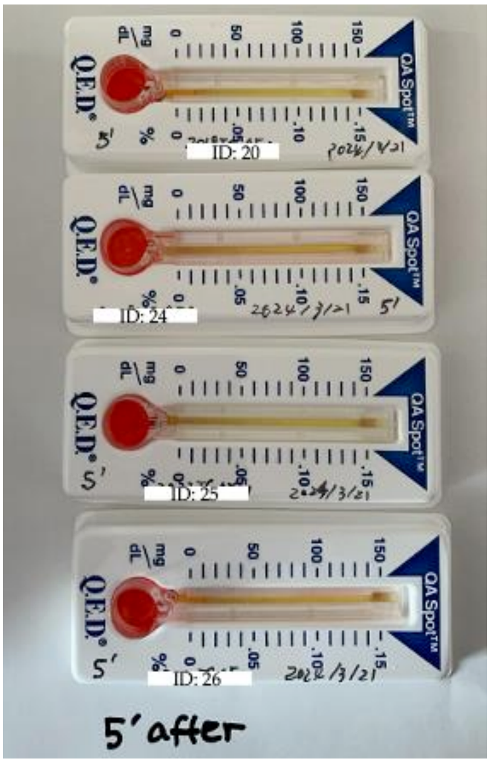

Figure S2 Five minutes after adding blood sample

Table S3. Lethal arrhythmia-evoked test results data bank

ID	Group	Result	Note
1	AL+SCD, Et ( )	Died during rearing	
2	AL+SCD, Et ( )	Negative	VPCs during ARS and Ep
3	AL+SCD, Et ( )	Negative	VPCs during ARS and Ep
4	AL+SCD, Et ( )	Positive	VT and CAVB during ARS, none during Ep
5	AL+SCD, Et ( )	Negative	VPCs during ARS and Ep
6	AL+SCD, Et ( )	Negative	VPCs during ARS and Ep
7	AL+SCD, Et ( )	Negative	SVPCs during ARS and Ep
8	AL+SCD, Et ( )	Negative	VPCs during ARS, none during Ep
9	AL+SCD, Et ( )	Died just before the lethal arrhythmia-evoked test	
10	AL+AD, Et ( )	Negative	VPCs during ARS and Ep
11	AL+AD, Et ( )	Negative	VPCs during ARS and Ep
12	AL+AD, Et ( )	Negative	VPCs during ARS and Ep
13	AL+AD, Et ( )	Negative	VPCs during Ep, none during ARS
14	AL+AD, Et ( )	Died during rearing	
15	AL+AD, Et ( )	Negative	VPCs during ARS and Ep
16	AL+AD, Et ( )	Positive	VT and CAVB during ARS and Ep
17	AL+AD, Et ( )	Negative	VPCs during ARS and Ep
18	AL+AD, Et ( )	Negative	VPCs during ARS and Ep
19	AL+AD, Et (+)	Positive	CAVB and VPCs during ARS, VT during Ep
20	AL+AD, Et (+)	Positive	CAVB during ARS, VT during Ep
21	AL+AD, Et (+)	Death	Died during ARS. CAVB and VT were observed.during ARS and Ep
22	AL+AD, Et (+)	Died the day before the end of the study	
23	AL+AD, Et (+)	Death	Died during ARS. CAVB and VT were observed. during ARS, VT during Ep
24	AL+AD, Et (+)	Positive	CAVB & VT during ARS and Ep
25	AL+AD, Et (+)	Positive	CAVB during ARS and Ep
26	AL+AD, Et (+)	Positive	CAVB & VT during ARS and Ep
27	WT+SCD, Et ( )	Negative	No abnormal findings
28	WT+SCD, Et ( )	Negative	No abnormal findings
29	WT+SCD, Et ( )	Negative	No abnormal findings
30	WT+SCD, Et ( )	Negative	No abnormal findings
31	WT+SCD, Et ( )	Positive	VT during Ep, none during ARS
32	WT+SCD, Et ( )	Negative	No abnormal findings
33	WT+SCD, Et ( )	Negative	No abnormal findings
34	WT+SCD, Et ( )	Negative	No abnormal findings

VPC: premature ventricular contraction; SVPC: supraventricular premature contraction

ARS: acute restraint stress; Ep: an intraperitoneal infusion of epinephrine

VT: ventricular tachycardia; CAVB: complete atrioventricular block
